# New Insights into the Relationships between Bacterial Dynamics and Water Quality of Aquaculture Systems Supplemented with Carbon Source and Biofilm Substratum

**DOI:** 10.3390/microorganisms9102168

**Published:** 2021-10-18

**Authors:** Er-Meng Yu, Zhen-Chi Li, Zhi-Fei Li, Guang-Jun Wang, Yun Xia, Kai Zhang, Jing-Jing Tian, Wang-Bao Gong, Jun Xie

**Affiliations:** 1 Guangdong Provincial Key Laboratory of Aquatic Animal Immune Technology, Pearl River Fisheries Research Institute of CAFS, Guangzhou 510380, China; boyem34@hotmail.com (E.-M.Y.); lzf@prfri.ac.cn (Z.-F.L.); gjwang@prfri.ac.cn (G.-J.W.); xy@prfri.ac.cn (Y.X.); zk@prfri.ac.cn (K.Z.); tianjj@prfri.ac.cn (J.-J.T.); gwb@prfri.ac.cn (W.-B.G.); 2 State Environmental Protection Key Laboratory of Environmental Pollution Health Risk Assessment, South China Institute of Environmental Sciences, Ministry of Ecology and Environment, Guangzhou 510655, China; lizhenchi@scies.org

**Keywords:** bacterial community, biofilm substrata, freshwater aquaculture, 16S rRNA amplicon, fish growth

## Abstract

Aquaculture is crucial for achieving the FAO’s goal of a world without hunger and malnutrition. Recently, biofilm substratum has been proposed as an effective means to control waste pollution caused by excessive nutrient inputs from aquaculture, but key bacterial communities involved in the remediation remain unclear. Here we reported a freshwater mesocosm study where the addition of biofilm substrata with external carbon effectively controlled the total ammonia nitrogen and improved fish growth. 16S rRNA study and Weighted UniFrac analysis revealed that bacterial compositions were significantly different (999 permutations, *p*-value < 0.01) between the biofilm-substrata-added and biofilm-substrata-free systems. Planctomycetes were found, as key bacteria benefited from the biofilm substrata addition and exerted the major function of ammonia nitrogen control. Our study demonstrated that the addition of biofilm substrata and an external carbon source favored fish growth and improved the aquaculture environment by the formation of a unique bacteria community.

## 1. Introduction

The annual global growth in fish consumption has been twice as high as population growth since 1961, demonstrating that fisheries are crucial in achieving the FAO’s goal of a world without hunger and malnutrition [1]. In 2016, aquaculture represented 47% of total global fish production, and 64.2% of these aquaculture activities occurred in freshwater systems [1]. Aquaculture also has a high potential to restore overfished stocks, as stated by the 2030 Agenda for Sustainable Development (2030 Agenda for short). In fact, the rapid development of aquaculture has been called the “blue revolution” because the huge amount of fish production contributes to human nutrition in a similar way as the “green revolution” [2]. However, the rapid global expansion of the aquaculture industry has caused many environmental issues, including waste pollution, eutrophication, ecosystem degradation, and disease outbreaks [3]. To establish sustainable aquaculture, it is important to develop resource-efficient farming systems that control pollution while maintaining desired fish growth [4].

Along with other various aquaculture wastes, aquafeed contributes to water pollution the most by disturbing aquatic communities [5]. As major habitants in aquatic systems, bacteria are essential for maintaining aquatic ecology [6,7]. Aquafeed is rich in protein, lipids, and other nutritional matters, of which 62% nitrogen and 70% phosphorus are released into the water [8]. The excessive nutrient inputs into the aquatic system agitate the inherent bacteria composition, further leading to water eutrophication [9]. Severe disturbance of bacterial community is difficult to recover [10], and the reduction of biodiversity and unexpected predominance of bacteria adversely affect the aquatic environment as a whole [11]. Given the rapid increase in global aquafeed, expected to rise to 58.85 million tons by 2020 and 73.15 million tons by 2025, respectively [12], the remediation of the bacterial community and maintenance of their ecological function is essential in the sustainable development of aquaculture.

The addition of biofilm substrata in conjunction with external carbon sources has been found to improve water quality and promote the growth of aquatic animals [13]. A previous study also found that biofilm substrata effectively reduced the ammonia content in water, which might be why the water quality was improved [14]. Biofilm substrata were found to increase the bacterial diversity, which served as a freshwater ecology restorer [15]. Although bacteria were believed to be the major participants in water quality changes, it remains unknown which key bacterial communities are responsible for the improvement of the aquatic environment. Here we present a mesocosm study on freshwater aquaculture systems to which biofilm substrata and external carbon sources were added. By combining the monitoring of aquatic environmental factors and the dynamic analyses of bacterial communities, we aimed to (i) identify the shift of bacterial communities in aquatic environment treated with external biofilm substratum and carbon source, (ii) investigate the key functional units during aquatic quality improvement.

## 2. Materials and Methods

### 2.1. Experiments and Reagents

#### 2.1.1. Mesocosm Systems Set-Up and Sample Collections

**Figure 1:**
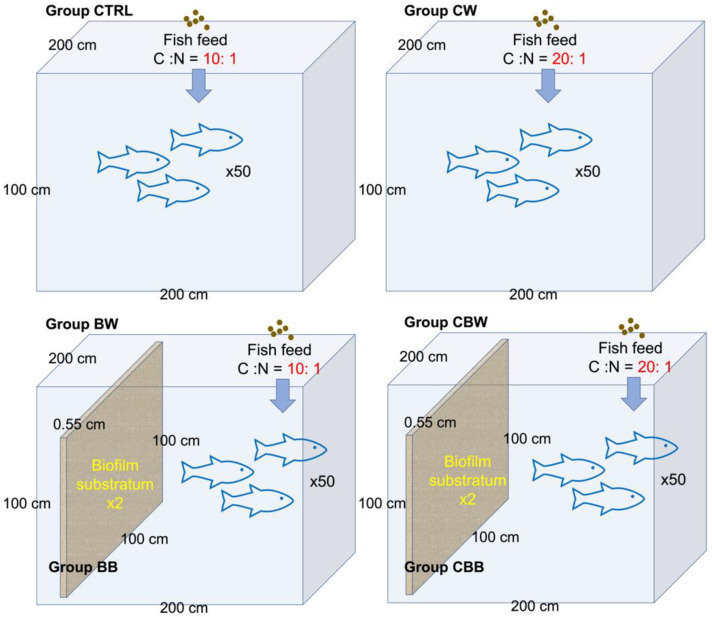
Set up of mesocosm systems. The top two cuboids represent the biofilm-substrata-free systems, while the bottom two are biofilm-substrata-added. The carbon source of the mesocosm systems was adjusted by the carbon and nitrogen ratio (C:N) of the fish feed. Four types of mesocosm systems were set up: C:N = 10:1 and biofilm-substrata-free system (top left, also severed as the control system); C:N = 20:1 and biofilm-substrata-free system (top right); C:N = 10:1 and biofilm-substrata-added system (bottom left); C:N = 20:1 and biofilm-substrata-added system (bottom right). CTRL, CW, BW, and CBW stand for the water samples of corresponding mesocosm systems, while BB and CBB stand for the biofilm-substrata samples.

This study was performed at the Aquaculture Base of Pearl River Fisheries Research Institute, Chinese Academy of Fisheries Science, Guangzhou, China. The experiment was continued for 60 days (d) in tanks that measured 2 m × 2 m × 1 m. The tanks were initially disinfected and aerated for 24 h/d. Source water originated from the Pearl River (23°4′7″ N, 113°13′3″ E) and was used to set up the closed mesocosm systems. A pumping system was applied on all the mesocosms for the aeration, and all the mesocosms were aerated for 10 d after the source water was added. Grass carp (120.0 ± 0.64 g) were stocked at a density of 50 individuals per tank and were fed twice a day. The carbon ratio of the systems was adjusted by the addition of different types of fish feed. For the systems of C:N = 10:1, formulated pellet feed containing 30% protein (Tongwei Company, Chengdu, China) with a C:N ratio close to 10:1 was applied. For C:N = 20:1, an external carbon source was applied to raise the C:N ratio (0.92 kg of D-glucose per kg of formulated feed). For the biofilm-substrata-added systems, two pieces of biofilm substrata (100 cm × 100 cm × 0.55 cm/piece, AquaMats^®^, Logan, UT, USA) was placed vertically in each tank and fixed on the bottom of the tank. The 2 substrata were placed 50 cm from each other. The AquaMats^®^ are constructed from fibers made of low density, condensed copolymers of polyethylene. The elucidation of the mesocosm systems is shown in Figure 1.

Four types of mesocosm systems were set up (Figure 1), and six types of samples were collected: the water samples of the control system (CTRL, C:N = 10:1 and biofilm-substrata-free); the water sample of the carbon-source-added and biofilm-substrata-free system (CW, C:N = 20:1 and biofilm-substrata-free); the water samples of the biofilm-substrata-added system (BW, C:N = 10:1 and biofilm-substrata-added) and the corresponding biofilm-substrata samples (BB); the water samples of the carbon-source-added and biofilm-substrata-added system (CBW, C:N = 20:1 and biofilm-substrata-added) and the corresponding biofilm-substrata samples (CBB). In addition, the source water (W0) at the beginning of the experiment was also collected.

Water samples of each mesocosm system were collected from three random locations of the tank and were pooled for analysis. Biofilm-substrata samples of each biofilm-substrata-added system were pooled from three pieces of cut biofilm substrata from different depths (25, 50, and 75 cm below the water’s surface). Water samples were collected every 10 days within a 60-day experiment for the measurement of water quality parameters. Both water samples and biofilm-substrata samples collected at the beginning (day 0), day 20, day 40, and day 60 were compiled for the bacterial community study.

#### 2.1.2. Water Quality Monitoring and Fish Growth

Weights (W) of the grass carp (*n* = 50) were recorded at both the beginning (Day 0) and the end (Day 60) of the experiment in all the mesocosm systems. Aquatic quality parameters were measured soon after the water samples were collected and transported to the laboratory. Temperature, dissolved oxygen (DO), and pH of water samples were measured by a YSI digital meter (Columbus, OH, USA). Total ammonia nitrogen (TAN), nitrate-nitrogen (NO_3_-N), nitrite-nitrogen (NO_2_-N), total nitrogen (TN), total phosphorus (TP), and active phosphate (PO_4_-P) of the water samples were measured according to the method described in the Chinese National Standard Methods GB/T 12763.4-2007. Chlorophyll-a content in the water samples was measured by the Chinese National Standard Method GB/T 12763.6-2007. Chemical oxygen demand (COD) was detected by a COD analyzer (HACH, Ames, IA, USA). Total phytoplankton was obtained using a plankton net (45-μm mesh) from 5 L of water collected and pooled from 5 different locations in each tank. Qualitative and quantitative estimations of the plankton were performed using a Sedgewick-Rafter (S-R) cell. All the above water quality parameters were measured every 10 days within a 60-day experiment.

#### 2.1.3. DNA Extraction and 16S rRNA Amplicon Sequencing

Water samples of 200 mL were collected and filtered with 0.2 µm membrane filters. The membrane filters were cut into small pieces and stored in a 50 mL centrifuge tube until DNA extraction. Cut pieces of biofilm substrata were added with 200 mL sterilized water and vibrated for 2 h. DNA extraction was conducted by a PowerFecal^TM^ DNA extraction kit (MO BIO Laboratories, Carlsbad, CA, USA). The primer was 515F/806R for 16S rRNA V4 region (515F: 5′-GTGCCAGCMGCCGCGGTAA-3′; 806R: 5′-GGACTACHVGGGTWTCTAAT-3′). Polymerase chain reaction (PCR) was conducted by Phusion^®^ High-Fidelity PCR Master Mix (NEB Ltd., Beijing, China) following the programming of 94 °C for 3 min; 35 cycles of 94 °C for 45 s, 56 °C for 45 s, 72 °C for 45 s; 72 °C for 10 min. The PCR products were collected, purified by Agencourt AMPure XP magnetic beads (Beckman Coulter, Brea, CA, USA), and were ready for sequencing. The sequencing was performed by MiSeq (Illumina, San Diego, CA, USA).

### 2.2. Data Processing and Statistical Analysis

In this study, four types of water samples and two types of biofilm-substrata samples were collected. Mesocosm systems were separated by their carbon source level (C:N = 10:1, C:N = 20:1). In all the statistical testing in this study, at each of the carbon source levels, water samples were grouped by whether their corresponding mesocosm systems were added with biofilm substrata (biofilm-substrata-added; biofilm-substrata-free). The biofilm-substrata samples were separated by the carbon source level of the mesocosm systems (C:N = 10:1; C:N = 20:1) for comparison.

#### 2.2.1. Measurement of Fish Growth and Water Quality

The grass carp growth condition in this study was measured by their weight gain rate (WGR). Grass carps in each of the mesocosm systems were weighted at the beginning and at the end of the experiment, the average weights were calculated (mean ±  sd). The WGR of grass carps (*n* = 50 per system) was calculated from the final and initial average weights: (*W*_Final_ − *W*_Initial_)/*W*_Initial_ × 100%. Grass carps WGR in the carbon-source-addition or/and the biofilm-substrata-addition system were compared to the control system. Feed conversion ratio (FCR) was calculated by total feed intake/(final weight − initial weight). For the water quality parameters, the differences in the water samples between biofilm-substrata-added systems and biofilm-substrata-free systems were contrasted. Wilcoxon Rank Sum test was applied to test the significances of the above differences.

#### 2.2.2. 16S rRNA Amplicon Data Processing and Taxonomic Classification

The outputted 16S rRNA data was imported into Quantitative Insights Into Microbial Ecology2 (QIIME2, v. 2020.8) [16] for data processing. Adaptors and primers of the sequences were trimmed. DADA2 [17] algorithm was applied for the sequences denoising, generating representative sequences and amplicon sequence variants (ASVs) table. SATé-enabled phylogenetic placement (SEPP) algorithm [18] was applied to generate a reliable phylogenetic tree against the 99% GreenGenes database (v. 13.8) [19]. ASVs table was rarefied to the minimum library size (20,279) before the diversity analysis. ASVs with a sampling frequency <5 were removed. Taxonomic classification was conducted by QIIME2 plugin feature-classifier and classify-sklearn algorithm against the 99% GreenGenes database (v. 13.8).

#### 2.2.3. Diversity Analysis

Chao1 index values of samples were calculated for the measurement of the bacterial alpha diversity [20]. For the water samples, sample alpha diversity between biofilm-substrata-added systems and biofilm-substrata-free systems were contrasted, while biofilm samples in two biofilm-substrata-added systems (different carbon source levels) were compared. The differences were tested by Welch’s *t*-test.

For the beta diversity, Weighted Unifrac [21] dissimilarity was calculated from the ASVs table, and the phylogenetic tree resulted from SEPP. Principal coordinate analysis (PCoA) was performed to visualize the results. Permutational multivariate analysis of variance (PERMANOVA) was applied for the testing of the significance of PCoA results [22].

#### 2.2.4. Relative Abundance of the Bacterial Community

The ASVs table was collapsed to the phylum level according to the taxonomic classification results. The relative abundance of the phyla in each sample was calculated by the total sum scaling. Phyla with a relative abundance <1% were assigned to “Others”.

#### 2.2.5. Bacterial Distinction

The ASVs table was collapsed to the latest classified level according to the taxonomic classification results. ANOVA-Like Differential Expression2 (ALDEx2) was applied to build the statistical model between water samples grouped by biofilm-substrata-added systems and the biofilm-substrata-free and between the biofilm-substrata samples in two biofilm-substrata-added systems with different carbon source levels and to calculate the effect size of bacteria in the samples [23]. The medium differences of bacteria between groups were also calculated and were tested by the Wilcoxon Rank Sum test. In the comparison between groups, bacteria with effect size threshold >1 and Benjamin-Hochberg (BH) adjusted *p*-value < 0.01 were denoted as significantly distinct bacteria.

#### 2.2.6. Genera Co-Occurrence Network

ASVs table was collapsed to genus level. Spearman’s correlation was calculated between genera in each of the mesocosm systems. In the biofilm-substrata-added systems, genera in both water samples and biofilm-substrata samples were pooled for the correlation calculation. Nodes with a Spearman correlation rho >0.6 and BH adjusted *p*-value < 0.05 were imported in the Cytoscape software (v. 3.8.2) for the network construction. The topological parameters (degree size, betweenness centrality, and closeness centrality) of nodes were calculated by NetworkAnalyzer [24]. Nodes were ranked by each of the parameters, and the network centralities were determined by the top average ranks of the nodes. Only nodes directly connected by the network centralities were shown.

## 3. Results

### 3.1. Fish Growth and Water Quality

#### 3.1.1. Fish Weight and WGR

Grass carps (50 per system) were weighted at Day 0 and Day 60 of the experiment. The average weights and WGR of grass carps in each mesocosm system are shown in Table 1. At the end of the experiment, the final weights (*W*_Final_) of grass carps in all the systems were higher than their initial weights (*W*_Initial_). At both carbon source levels, compared with those in the biofilm-substrata-free systems, the WGR of grass carps were higher in the biofilm-substrata-added systems, where the WGR was the greatest in the system of C:N = 20:1 and biofilm-substrata-added while the lowest was found in the system of C:N = 20:1 and biofilm-substrata-free (Table 1).

#### 3.1.2. Water Quality Parameters

**Figure 2:**
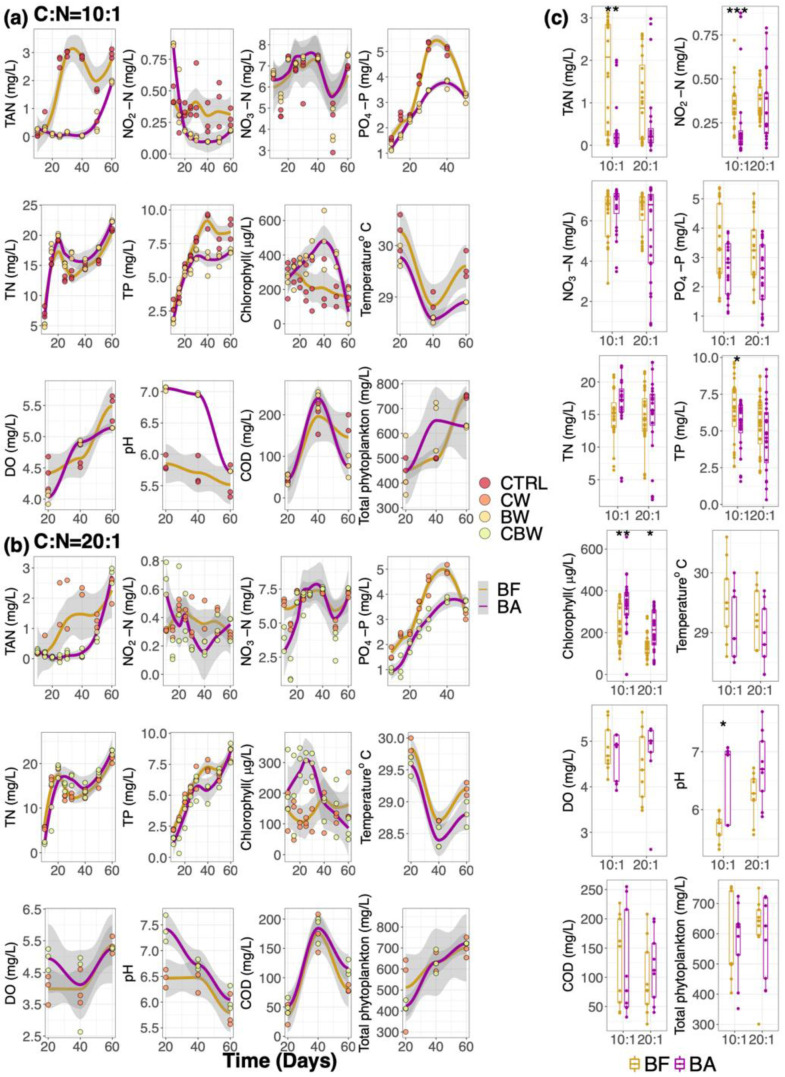
Water quality parameters in different mesocosm systems. Scatter plots showing the water quality parameters at different sampling time at carbon source level of (**a**) C:N = 10:1; (**b**) C:N = 20:1. Orange and purple lines are the Loess regression fit lines showing the trends of the water quality parameters changes in the biofilm-substrata-free system (BF) and biofilm-substrata-added system (BA). (**c**) Box plot showing water quality parameters contents. At both of the carbon source levels, samples were grouped by BF or BA. The differences of water quality parameters between BF and BA group were tested by Wilcoxon Rank Sum test (* *p*-value < 0.05, ** *p*-value < 0.01, *** *p*-value < 0.001).

Temperature, dissolved oxygen (DO), pH, total ammonia nitrogen (TAN), nitrate-nitrogen (NO_3_-N), nitrite-nitrogen (NO_2_-N), total nitrogen (TN), total phosphorus (TP), active phosphate (PO_4_-P), chemical oxygen demand (COD), total phytoplankton, and chlorophyll content of water samples in all the mesocosm systems were measured (Table S1, Supplementary File). Figure 2a,b shows the water quality parameters and their trends (Loess regression) in mesocosm systems at C:N = 10:1 and C:N = 20:1, respectively. At both carbon source levels, water quality parameters were grouped by biofilm-substrata-added or by biofilm-substrata-free, and their differences were tested by the Wilcoxon Rank Sum test (Figure 2c).

When the carbon source level was C:N = 10:1, TAN contents increased at the beginning of the experiment in the biofilm-substrata-free system. In contrast, TAN contents in the biofilm-substrata-added system remained constantly lower before day 40 but increased at day 60. NO_2_-N contents in the biofilm-substrata-added systems dropped dramatically from the beginning of the experiment. For PO_4_-P and TP contents, both of them increased at the beginning of the experiment and peaked at day 40. However, the Loess regression line for them showed that the overall and peak contents in the biofilm-substrata-added system were lower than that in the biofilm-substrata-free. Chlorophyll-a in the biofilm-substrata-free system dropped gradually throughout the experiment, whereas it increased and peaked at day 40 in the biofilm-substrata-added system (Figure 2a). Other water quality parameters shared a similarly changing pattern in both biofilm-substrata-added and biofilm-substrata-free at carbon source level of C:N = 10:1.

When the carbon source level was C:N = 20:1, water quality parameters in both the biofilm-substrata-added system and biofilm-substrata-free system changed in the same manner as at the carbon source level of 10:1 (Figure 2b).

For the overall contents, when the carbon source level was C:N = 10:1, TAN (*p*-value < 0.001), NO_2_-N (*p*-value < 0.001), TP (*p*-value < 0.05) contents in the biofilm-substrata-added system were significantly lower than them in the biofilm-substrata-free, while the chlorophyll content and pH of water were significantly higher. When the carbon source level was C:N = 20:1, only the chlorophyll content was significantly higher in the biofilm-substrata-added system compared with the biofilm-substrata-free (Figure 2c).

### 3.2. Bacterial Community in Water and Biofilm-Substrata Samples

#### 3.2.1. 16S rRNA Data

In this study, the 16S rRNA V4 region of bacteria in water and biofilm substrata was sequenced by Illumina MiSeq pair-end sequencing. In total, 1,772,947 reads were generated from 57 samples. After the DADA2 denoising, 1,420,127 high-quality and non-chimeric reads were retained. The reads utilization rate ranged from 73.22% to 90.09%. The numbers of ASVs were 4933. ASVs table was rarefied to minimum library size (20,279) before the diversity analysis. The statistics of raw sequence data and DADA2 denoising is shown in Table S2, while the ASVs table is shown in Table S3 of the Supplementary File.

#### 3.2.2. Alpha Diversity and Beta Diversity Analysis

**Figure 3:**
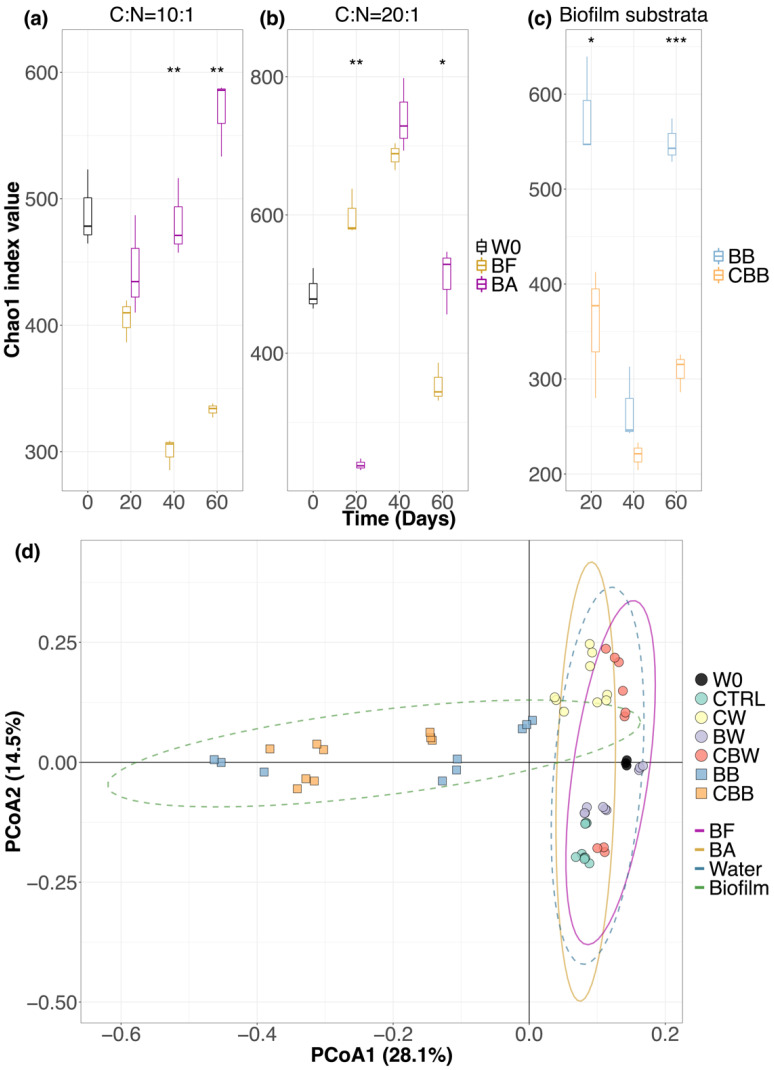
Diversity analysis of the bacterial community in water and biofilm-substrata samples. Chao1 index values of water samples in the biofilm-substrata-free system (BF) and biofilm-substrata-added system (BA) at (**a**) carbon source level of C:N = 10:1; (**b**) carbon source level of C:N = 20:1; and (**c**) the values of biofilm-substrata samples at two carbon source levels. W0 stands for the water samples of the source water. The differences of the Chao1 values between BF and BA at different time points were tested by Welch’s *t*-test. (**d**) Weighted UniFrac based principal coordinate analysis score plot. All samples were grouped by their sample type (water samples: blue dashed line ellipse; biofilm-substrata sample: green dashed line ellipse), while water samples were grouped by BF or BA (BF: purple solid line ellipse; BA: yellow solid line ellipse). The difference between groups was tested by the PERMANOVA test (* *p*-value < 0.05, ** *p*-value < 0.01, *** *p*-value < 0.001).

For the alpha diversity, the Chao1 index calculated the bacterial richness of water and biofilm-substrata samples (Table S4, Supplementary File). For the water samples, when the carbon source level was C:N = 10:1, the bacterial richness in the biofilm-substrata-added system raised continuously from day 20, whereas the bacterial richness in the biofilm-substrata-free system dropped and bottomed at day 40 (Figure 3a). The Chao1 index value was significantly higher in the biofilm-substrata-added system compared with that in the biofilm-substrata-free at day 40 (*p*-value < 0.01) and day 60 (*p*-value < 0.01). When the carbon source level was C:N = 20:1, the changes of bacterial richness in both the biofilm-substrata-added system and biofilm-substrata-free system were the same in that the values increased sharply from day 20 and peaked at day 40, but dropped at day 60 (Figure 3b). The Chao1 index value in the biofilm-substrata-added system was significantly lower at day 20 but higher at day 60 compared with that in the biofilm-substrata-free system. On the other hand, for the biofilm-substrata samples, the changes of the bacterial richness were similar at both carbon source levels, where the bacterial richness dropped from day 20 and bottomed at day 40 but increased dramatically at day 60 (Figure 3c). Nevertheless, the bacterial richness in the biofilm-substrata samples was generally higher when the carbon source level of C:N = 10:1 and was significantly higher at day 20 (*p*-value < 0.05) and day 60 (*p*-value < 0.001) compared with that when the carbon source level of C:N = 20:1.

Weighted UniFrac based Principal coordinate analysis (PCoA) was applied to study the beta diversity of the bacterial community of water samples and biofilm-substrata samples (Table S5, Supplementary File). The PERMANOVA test was performed to test the differences of beta diversity of the bacterial communities in samples by grouping them into different sample types (water samples or biofilm-substrata samples) for all the samples and into biofilm-substrata-added (BA) or biofilm-substrata-free (BF) for the water samples. The test results are shown in Table S6 in the Supplementary File. Figure 3d shows the clustering of samples that obvious separation was found between water samples and biofilm-substrata samples with significance (q-value < 0.01). In contrast, the separation between BA samples and BF samples was less obvious; nevertheless, the PERMANOVA test showed that the separation was significant (q-value < 0.01), which indicated that the bacterial composition of water samples in the biofilm-substrata-added system was significantly different compared with that in the biofilm-substrata-free.

#### 3.2.3. Bacterial Composition at Phylum Level

**Figure 4:**
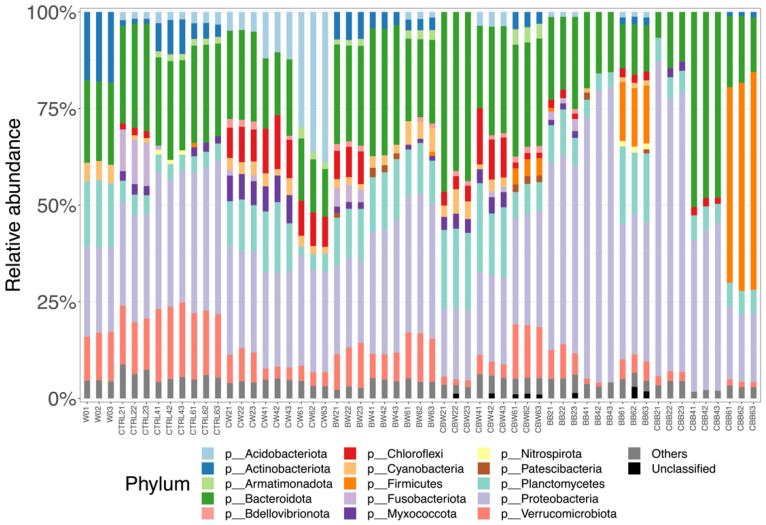
Bacterial compositions of water and biofilm samples in different mesocosm systems at the phylum level. Phyla with relative abundance < 1% were summed up and classified to “Others”. W0: source water; CTRL: water samples of C:N = 10:1 and biofilm-substrata-free system; CW: water samples of C:N = 20:1 and biofilm-substrata-free system; BW, BB: water samples, biofilm-substrata samples of C:N = 10:1 and biofilm-substrata-added system; CBW, CBB: water samples, biofilm-substrata samples of C:N = 20:1 and biofilm-substrata-added system.

The relative abundance of phyla was calculated and shown in Table S7 of the Supplementary File. At phylum level (Figure 4), bacterial composition of the source water (W0) was dominated by Proteobacteria (22.5 ± 0.67), followed by Bacteroidota (Bacteroidetes) (21.05 ± 0.40%), Actinobacteria (17.99 ± 0.33%), Planctomycetes (16.88 ± 0.49%) and Verrucomicrobiota (12.19 ± 0.72%). In water samples, Proteobacteria and Bacteroidota (Bacteroidetes) were the two most dominant phyla; their average relative abundances ranged from 23.04–33.02% and 18.43–32.49% in all the mesocosm systems. At carbon source level of C:N = 10:1, in addition to Proteobacteria and Bacteroidota (Bacteroidetes), Verrucomicrobiota (16.56 ± 2.26%) highly occupied the biofilm-substrata-free system whereas Planctomycetes (13.21 ± 1.46) moderately dominated in the biofilm-substrata-added system. On the other hand, when the carbon source level was C:N = 20:1, considerable amounts of Acidobacteriota (17.13 ± 13.9%) and Planctomycetes (10.45 ± 5.55%) were found in the biofilm-substrata-free system, whereas Planctomycetes (15.5 ± 6.43%) and Verrucomicrobiota (6.48 ± 5.48%) were abundant in the biofilm-substrata-added system (Figure 4). On the other hand, similar to the phyla composition of water samples in the biofilm-substrata-added systems, the majority phyla in biofilm samples were Proteobacteria (BB: 52.63 ± 16.61; CBB: 44.75 ± 24.87%) and Bacteroidota (Bacteroidetes) (BB: 16.86 ± 4.38%; CBB: 25.61 ± 17.77%). However, the subsequently dominant phyla in biofilm samples differed from the water samples, which were composed of Planctomycetes (BB: 10.52 ± 6.15%; CBB: 5.85 ± 0.44%) and Firmicutes (BB: 5.15 ± 7.49%; CBB: 17.86 ± 26.83%). The additional information of the bacterial composition at the family level is shown in Table S8 of the Supplementary File.

#### 3.2.4. Bacterial Distinction

**Figure 5:**
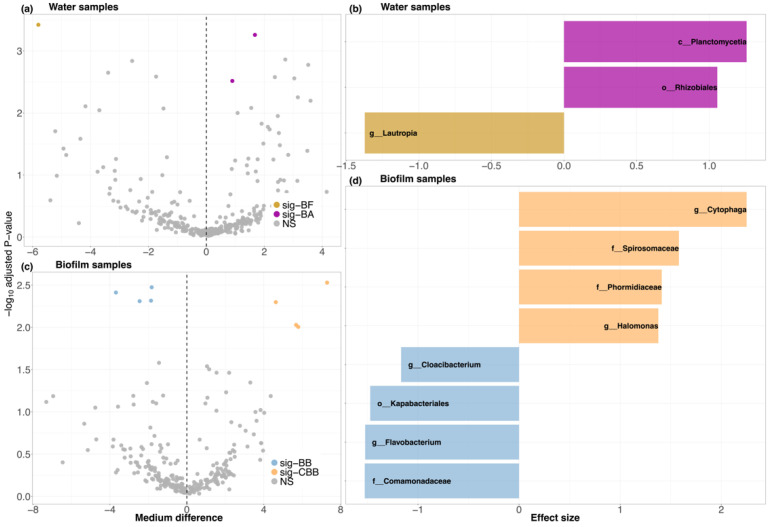
ALDEx2 test results showing the distinct ASVs at their latest classified level. (**a**) Volcano plot of bacteria in water samples between biofilm-substrata-added (BA) and biofilm-substrata-free (BF) systems, and (**b**) bar plots of the significant distinct bacteria between BA and BF. (**c**) Volcano plots of bacteria in biofilm-substrata samples between C:N = 10:1 and C:N = 20:1 systems, and (**d**) the corresponding distinct bacteria between the groups. In all the compassions, bacteria with effect size threshold > 1 and adjusted *p*-value < 0.01 were defined as significant district bacteria and were colored in the plots.

ASVs were collapsed to their latest classified level before the ALDEx2 analysis (Table S9). Water samples were grouped by whether their systems were added with biofilm-substrata, while biofilm-substrata samples were grouped by the carbon source levels of the systems. Water samples and biofilm-substrata samples were tested separately by the ALDEx2 statistical model (Table S10 showed the test results). Taxa with adjusted *p*-value < 0.01 and effect size threshold > 1 were defined as distinct bacteria. In water samples, class Planctomycetia and order Rhizobiales were significantly distinct in biofilm-substrata-added systems, whereas genus *Lautropia* was distinct in the biofilm-substrata-free (Figure 5a,b). On the other hand, in the comparison between biofilm samples, families of Spirosomaceae, Phormidiaceae and between *Cytophaga*, *Halomonas* were significant when the carbon source level was C:N = 20:1, while order Kapabacteriales, family Comamonadaceae and genera of *Cloacibacterium* and *Flavobacterium* were significant at C:N = 10:1 (Figure 5c,d).

#### 3.2.5. Genera Co-Occurrence Network

**Figure 6:**
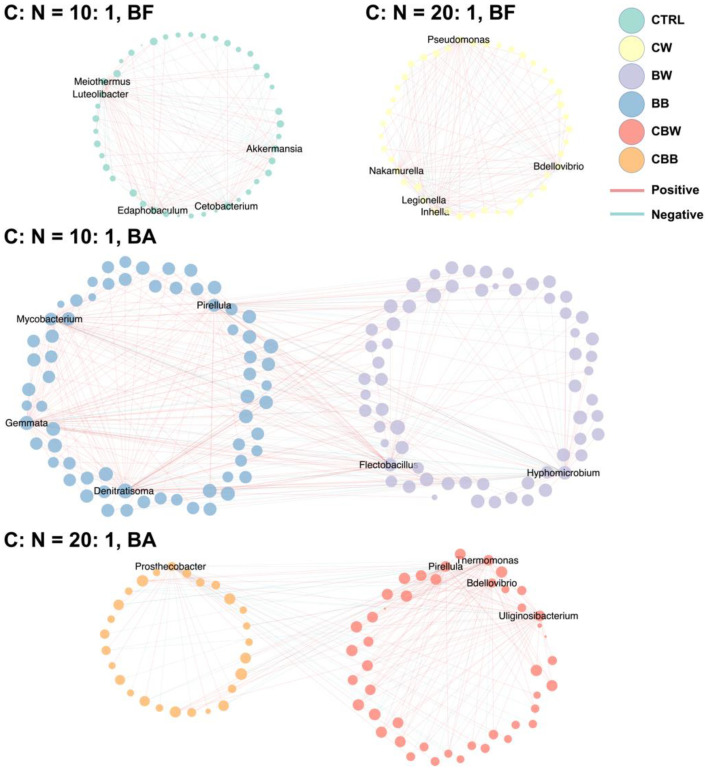
Genera co-occurrence network in mesocosm systems. Spearman correlation was calculated between genera in each of the systems. In the biofilm-substrata-added (BA) systems, genera in water samples and biofilm samples were pooled for the calculations. Nodes in different colors are genera in samples of CTRL: water samples of C:N = 10:1 and biofilm-substrata-free system (top left); CW: water samples of C:N = 20:1 and biofilm-substrata-free system (top right); BW, BB: water samples, biofilm-substrata samples of C:N = 10:1 and biofilm-substrata-added system (middle); CBW, CBB: water samples, biofilm-substrata samples of C:N = 20:1 and biofilm-substrata-added system (bottom). The size of nodes is proportional to the degree size of the nodes. Edges in red and green represent the positive correlation and negative correlation, respectively. Network centralities in each of the systems were labeled.

In order to study the bacterial co-occurrence pattern in different mesocosm systems, the ASVs table was collapsed to the genus level (Table S11). Spearman’s correlations of genera in each of the systems were calculated (in biofilm-substrata-added systems, genera in water samples and biofilm-substrata samples were pooled). Pairs of genera with a Spearman correlation rho >0.6 and BH adjusted *p*-value < 0.05 were imported in the Cytoscape software for the network construction. Consequently, 66, 65, 152 and 104 genera met the criteria and were constructed for the networks in systems of C:N = 10:1 and biofilm-substrata-free (BF) system, C:N = 20:1 and BF system, C:N = 10:1 and biofilm-substrata-added (BA) system and C:N = 20:1 and BA system, respectively (Table S12). The topological parameters (degree size, betweenness centrality, and closeness centrality) of nodes in each network were calculated by NetworkAnalyzer to determine the network centralities (Table S12). In the biofilm-substrata-free systems, when the carbon source level was C:N = 10:1, *Akkermansia*, *Cetobacterium*, *Edaphobaculum*, *Luteolibacter,* and *Meiothermus* were determined as network centralities while *Bdellovibrio*, *Inhella*, *Legionella*, *Nakamurella*, and *Pseudomonas* centered the bacterial network when the carbon source level was C:N = 20:1. In the biofilm-substrata-added systems, when the carbon source level was C:N = 10:1, *Hyphomicrobium* and *Flectobacillus* from water samples as well as *Denitratisoma*, *Gemmata*, *Mycobacterium*, *Pirellula* from biofilm-substrata samples were found as network centralities. In contrast, when the carbon source level was C:N = 20:1. *Pirellula*, *Thermomonas*, *Bdellovibrio*, and *Uliginosibacterium* in water samples together with *Prosthecobacter* in biofilm-substrata samples centered at the network (Figure 6).

## 4. Discussion

### 4.1. Biofilm Substratum Shifted the Predominance of Bacteria in Mesocosm Aquaculture Systems

In this study, the Weighted UniFrac results showed that bacterial compositions in BA and BF systems were significantly different. A more obvious difference was found between the water samples and biofilm-substrata samples, which implied that the bacterial composition in the water interface and biofilm-substrata interface was entirely different. Therefore, it was suspected that the bacteria on the biofilm-substrata interface might contribute to the variation of bacteria in the water interface. However, the interaction of bacteria between water and biofilm-substrata interfaces requires further confirmation.

In this study, the relative abundance of Acidobacteriota (Acidobacteria) was found to increase dramatically along with the time in the system of C:N = 20:1 and biofilm-substrata-free (BF). Additional glucose was supplemented in the fish feed to increase the overall carbon source level of the C:N = 20:1 systems in this study. The carbon source level in these systems might be increased in two ways: direct diffusion of fish feed into the water; and the metabolism and excretion by fish; bacteria in the systems could utilize the carbon source in both ways. A previous study showed that Acidobacteriota (Acidobacteria) preferred glucose as a carbon source [25], which could explain the dramatic growth and predominance of Acidobacteriota (Acidobacteria) in the C:N = 20:1 and BF system. However, at the same carbon source level, when the system was biofilm-substrata-added (BA), the predominant bacteria changed from Acidobacteriota (Acidobacteria) to Bacteroidota (Bacteroidetes). In the C:N = 20:1 and BA system, biofilm substrata were added, which offered additional surfaces for bacteria to adhere to and grow. The previous finding of gene coding for surface adhesion protein in Bacteroidota (Bacteroidetes) indicated its strong adhesion ability [26]. It is therefore suggested that the replacement of Acidobacteriota (Acidobacteria) by Bacteroidota (Bacteroidetes) may be attributed to the biofilm-substrata addition because Bacteroidota (Bacteroidetes) preferred to grow on an interface, with less energy consumed compared with growing while floating [26].

### 4.2. Biofilm Substratum Addition Controlled Ammonia Nitrogen and Benefited Fish Growth

Our results showed that the addition of biofilm substrata significantly increased the WGR of grass carp in mesocosm aquaculture systems at both of the carbon source levels. In this study, the WGR of grass carp was the greatest in the mesocosm system with a carbon source level of C:N = 20:1 and biofilm-substratum-added (BA), followed by that in the system of C:N = 10:1 and BA, which were approximately 1.5 times higher than the WGR of grass carp in the system of C:N = 10:1 and biofilm-substratum-free (BF) and 6.6 times higher than that in the system of C:N = 20:1 and BF. Compared with the BF systems, we found that the contents of total ammonia nitrogen (TAN) in BA systems were significantly lower. TAN was found to adversely affect grass carp growth even at low concentrations [27]. Therefore, the lower WGR of grass carp in the BF systems might be linked with their corresponding higher TAN contents. Parallel to the findings of grass carp WGR in different systems, bacterial richness in water samples was generally higher in the BA systems compared with the BF systems. Previous studies have demonstrated the interactions of bacteria between the water and biofilm-substrata interfaces [14,28]; the bacterial richness in water samples of BA systems in this study is therefore believed to be supplemented by the biofilm-substrata interface. The higher bacterial richness in BA systems indicated stronger activities of bacteria which implied that the consumption of carbon sources and nitrogen sources might be fast. As mentioned in Section 4.2 in this study, at the high carbon source level (C:N = 20:1), the predominance of Acidobacteriota (Acidobacteria) was replaced by Bacteroidota (Bacteroidetes) when the biofilm substrata were added. Some Bacteroidota (Bacteroidetes) species were reported to have genes for ammonium assimilation [29], which indicated their ability of total ammonia control. Nevertheless, the control system (C:N = 10:1 and BF) predominated by Bacteroidota (Bacteroidetes) was found rich in TAN contents, which indicated that detail species or strain level of Bacteroidota (Bacteroidetes) required further identifications to confirm their role of TAN removal in the systems. On the other hand, the relative abundance of Planctomycetes in BA systems was higher than that in BF systems in this study. In addition, the ALDEx2 test result showed that Planctomycetia, a class from lineage of phylum planctomycetes, was significantly distinct in BA systems. Planctomycetes are mostly anammox bacteria that convert ammonia and nitrite into nitrogen gas through anoxic ammonia oxidization [30]. The abundance and distinction of Planctomycetes in BA systems implied their preference for biofilm substrata addition, which strongly suggested their roles of major TAN controller. Moreover, *Pirellula* (genus from lineage Planctomycetes) was found as network centralities in BA systems, which implied that Planctomycetes were also the bacterial keystones in the BA systems and dominated the bacterial activities in the BA systems.

However, the TAN contents were raised at day 60 at most of the systems. Especially when the carbon source level was high, the TAN contents in the BA systems were even greater than that in the BF system. Interestingly, at the carbon source level of C:N = 20:1, bacterial richness dropped dramatically at day 60, which was suspected to be the reason for the increase of TAN contents in water. The excessive growth of bacteria in a limited space might lead to the collapse of the environment and raise the ammonia nitrogen contents.

In addition to the TAN contents, the chlorophyll-a content in the mesocosm systems was another possible reason affecting the grass carp WGR. In this study, the chlorophyll-a contents in BA systems were significantly higher than that in the BF systems, which indicated that the number of algae was comparatively abundant when the biofilm-substrata were present. Algae might serve as an additional food source for grass carp and therefore increased the WGR of the fishes. However, further studies on the mechanism of algae growth promoted by the BA systems are required to prove the results.

Our study applied the 16S rRNA amplicon study on the bacteria of the mesocosm systems, which was limited in the determinations of the detailed taxonomic classification of the bacteria and their key functional genes in the systems. In a future study, metagenomics or other whole-genome sequencing techniques should be used to further verify the results in this study.

## 5. Conclusions

Our study applied the 16S rRNA study on the bacterial community dynamics in mesocosm aquaculture systems supplemented with biofilm substrata and external carbon sources. We found that the grass carp weight gain was significantly increased when the systems were biofilm-substrata-added compared with those of biofilm-substrata-free. Bacteroidota (Bacteroidetes) favored the high carbon source level, but its dominance was replaced by Acidobacteriota (Acidobacteria) when biofilm substrata were added to the systems. Planctomycetes were found as potential key bacteria in controlling total ammonia nitrogen in the biofilm-substrata-added systems. Our findings are significant for the establishment of sustainable aquaculture. In a further study, metagenomics or targeted sequencing technique is needed to identify further the strain level of the bacteria in this study.

## Figures and Tables

**Table 1:** Grass carps weight gain rate in different mesocosm systems.

Mesocosm Systems	Water Samples	Biofilm Substrata Samples	Carbon Source Level (C:N)	Biofilm Substrata	*W*_Initial_	*W*_Fianl_	WGR (%)	FCR
1	CTRL		10:1	Free	120.1 ± 1.8	187.1 ± 27.7	33.86 ± 11.65	3.35 ± 0.08
2	CW		20:1	Free	120.4 ± 0.9	130.3 ± 8.3	7.82 ± 4.92 ***	2.87 ± 0.17 ***
3	BW	BB	10:1	Added	120.6 ± 1.1	221.7 ± 31.2	43.93 ± 8.32 ***	3.10 ± 0.11 ***
4	CBW	CBB	20:1	Added	120.2 ± 1.4	252.5 ± 41.9	52.04 ± 7.81 ***	2.21 ± 0.14 ***

Notes: WGR, weight gain rate; FCR, feed conversion rate. *** Welch’s *t* test *p*-value < 0.001 compared with the CTRL group.

## Data Availability

The 16S rRNA amplicon data is available at NCBI, accession numbers: PRJNA737201. Available online: https://www.ncbi.nlm.nih.gov/bioproject/PRJNA737201 (accessed on 13 Junuary 2021).

## Supplementary Materials

The following are available online at https://www.mdpi.com/article/10.3390/microorganisms9102168/s1, Table S1: Water quality parameters, Table S2: Statistics of raw sequence data and DADA2 denoising, Table S3: ASVs table, Table S4: Chao1 index values of samples, Table S5: Weighted UniFrac based PCoA scores, Table S6: PERMANOVA test results, Table S7: Relative abundance of phyla, Table S8: Relative abundance of Families, Table S9: ASVs table at the latest classified level, Table S10: ALDEx2 test results, Table S11: ASVs table at the genus level, Table S12: Topological parameters of genera in bacterial co-occurrence network.
